# Endocannabinoid System in Pregnancy Maintenance and Labor: A Mini-Review

**DOI:** 10.3389/fendo.2021.699951

**Published:** 2021-06-16

**Authors:** Melissa L. Kozakiewicz, Chad A. Grotegut, Allyn C. Howlett

**Affiliations:** ^1^ Department of Obstetrics and Gynecology, Section on Maternal-Fetal Medicine, Wake Forest School of Medicine, Winston-Salem, NC, United States; ^2^ Department of Physiology and Pharmacology, Wake Forest School of Medicine, Winston-Salem, NC, United States

**Keywords:** endocannabinoid system, myometrium, labor, parturition, anandamide, cannabinoid receptor, preterm labor, pregnancy

## Abstract

The endocannabinoid system (ECS) is a cell-signaling system present in multiple organ systems and is an integral part of sustaining the microenvironment necessary for early pregnancy success and maintenance. It plays a significant role in embryo development, transport and implantation as well as placentation. The current theory behind the initiation of term labor is that it is a complex, multifactorial process involving sex steroid hormones, prostaglandin production and interplay at the maternal-fetal interface resulting in increased expression of receptors and gap junctions that promote uterine activation. There is increasing evidence that, in addition to early pregnancy events, the ECS plays a regulatory role in pregnancy maintenance and the timing of labor. This review presents an overview of the ECS in pregnancy that focuses on late gestation and parturition.

## Introduction

The endocannabinoid cell-signaling system (ECS) is based upon eicosanoid derivatives that promote cellular return to homeostasis in multiple organ systems and modulates smooth muscle function, metabolism of tissues, and immune function (1). The ECS includes CB_1_ (CB_1_R) and CB_2_ (CB_2_R) cannabinoid receptors, the endocannabinoid agonists anandamide (AEA) and 2-arachidonoylglycerol (2-AG), and the enzymes that synthesize and metabolize the endocannabinoid ligands (1). CB_1_R is a G protein-coupled receptor encoded by the CNR1 gene, the activation of which couples predominantly to Gα_i/o_ proteins to promote effects on calcium channels, mitogen-activated protein kinases (MAPKs) and adenylyl cyclase (2, 3). Cannabinoid receptor interacting protein 1a (CRIP1a) is a CB_1_R-associated protein that modulates trafficking of newly synthesized CB_1_Rs to the cell surface and attenuates receptor internalization (4).

Alterations in ECS signaling have been associated with early pregnancy loss (5). As recently reviewed, evidence supports significant contributions of the ECS in early pregnancy events including embryo transport, embryo implantation and placentation (6–8). There is also evidence supporting interplay among the sex steroid hormones, estrogen and progesterone, and the ECS (9, 10). Furthermore, inflammatory conditions in reproduction, including preeclampsia, miscarriage and endometriosis, have been associated with aberrant ECS signaling (11).

Pregnancy is considered a progesterone-dominant state, as progesterone is the major steroid hormone that contributes to the maintenance of pregnancy (12). Prior to the onset of labor, the uterus converts from a quiescent state to an active contractile state. The quiescent phase is maintained by progesterone and other factors that regulate contractile gene expression. In late pregnancy, there is an increase in estrogen and, with a growing fetus, an increase in myometrial stretch. This leads to increased expression of genes and receptors required for uterine contractions including prostaglandins (PG), connexin 43, and oxytocin receptor (OTR). Labor is a regulated inflammatory event during which prostaglandins PGF_2α_ and PGE_2_ contribute to cervical ripening and enhance uterine contractions. Timing of normal labor requires communication between the fetal and maternal units. A similar pathway to labor is apparent in patients with preterm labor, although, the etiology and phenotype of preterm labor differs (13). This review will focus on the influence of the ECS on pregnancy maintenance and the timing of labor.

## Endocannabinoid Influence in Pregnancy and Labor

**Figure 1:**
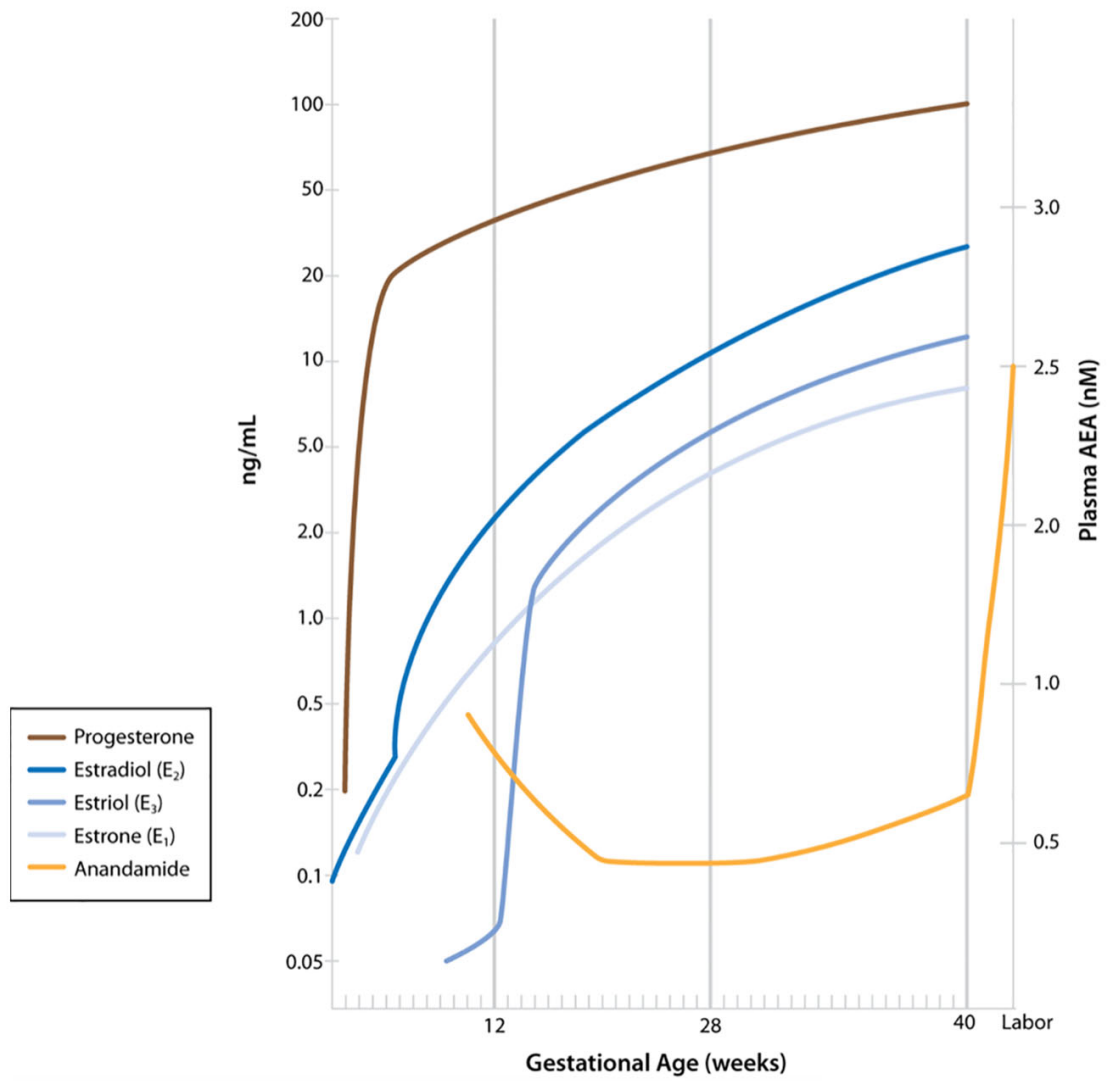
Maternal plasma anandamide levels as described by Habayeb and colleagues (14) relative to levels of progesterone and estrogens during pregnancy. (Figure including levels of progesterone and estrogens published in Boron WF, Boulpaep EL: Medical Physiology, 3^rd^ ed. Philadelphia, Elsevier, 2017. Data from Wilson JD, Foster DW, Kroenber M, Larsen PR: Williams Textbook of Endocrinology, 9^th^ ed. Philadelphia, WB Saunders, 1998. Used with permission.)

Several research groups have evaluated plasma AEA levels in pregnancy and in labor, reporting that plasma levels of AEA are predictive of the onset of parturition (14, 15). AEA is synthesized by *N-*acylphosphatidylethanolamine-specific phospholipase D (NAPE-PLD), as are other fatty acid ethanolamides such as oleoylethanolamide (OEA) and palmitylethanolamide (PEA). Each of these are degraded primarily by fatty acid amide hydrolase (FAAH). Of these, only AEA is an agonist for the cannabinoid receptors, and AEA can be oxidized by cyclooxygenase-2 (COX-2) to PG-ethanolamides (prostamides) (16). AEA levels have been shown to be higher in the estrogen-dominant phases than in the more progesterone-dominant phases of the menstrual cycle (14). Luteal phase AEA levels are similar to those in the first trimester of a successful pregnancy (14). In the progesterone-dominant state of pregnancy, plasma AEA levels decrease in the second and third trimesters (**Figure 1**) (14). Plasma AEA levels increase just prior to the onset of labor, followed by a significant increase in labor (14). The source of this AEA is postulated to be a response of the endothelial cells to estradiol (17). **Figure 1** shows AEA fluctuations as described along with the relative changes in progesterone and estrogen throughout pregnancy.

In addition to spontaneous labor, plasma AEA levels have been evaluated in induced labor (15). Nallendran and colleagues evaluated the percentage change in plasma AEA from a non-laboring to laboring state and determined AEA influence on induction-to-delivery interval (15). Their longitudinal observational study of 64 women showed a 1.5-fold increase in plasma AEA levels in labor. Furthermore, higher percentage rises in AEA were associated with shorter induction-to-delivery intervals.

Attempts to identify a marker that is reliably predictive of preterm delivery have been made, but current testing methods remain limited. The current standard of care for predicting preterm birth includes sonographic measurement of cervical length along with assessment of cervicovaginal fetal fibronectin (18). However, the positive predictive value of these tests is not ideal, as at least half of all women admitted for preterm labor end up delivering at term (18–20). The obstetric community remains in search of a non-invasive, reliable test to predict preterm birth. Bachkangi and colleagues investigated plasma levels of AEA, OEA, and PEA as potential markers of spontaneous preterm birth (21). They found plasma levels of AEA and PEA better predicted preterm birth in a high-risk population of women than the current standard methods. Additionally, plasma AEA levels predicted the gestational age of delivery.

AEA has been correlated with the expression of OTR in human placentas (22). Cells isolated from human placentas collected at term were cultured with methanandamide (meth-AEA). OTR mRNA was increased in the placental cells exposed to meth-AEA, and oxytocin concentrations were higher in the culture medium. These researchers proposed that AEA contributes to OTR expression and oxytocin release in labor (22). Yulia and colleagues investigated human primary myometrial cells isolated from uterine samples obtained at the time of cesarean section to determine the effect of cyclic adenosine monophosphate (cAMP)/protein kinase A (PKA) function on OTR expression (23). They identified an overall reduction in the cAMP/PKA pathway in late gestation and in labor to be associated with an increase in OTR mRNA and protein.

In ULTR myometrial cells, AEA-stimulated CB_1_R coupled to Gα_i/o,_ inhibiting adenylyl cyclase and decreasing intracellular cAMP (24). CB_1_R also mediated a time- and concentration-dependent increase in ERK phosphorylation (24). Exposure to high levels of AEA decreased cell numbers and changed morphology typical of apoptotic cell death, which could be prevented by a CB_1_R antagonist or MAPK inhibitor (24). This AEA- and CB_1_R-mediated, ERK-dependent reduction in cell viability suggests a potential physiologic relevance of AEA in decidual senescence and in postpartum uterine involution. AEA is known to act not only on CB_1_R, but also other receptors including the ligand-gated transient receptor potential vanilloid receptor type 1 (TRPV_1_) (25) and peroxisome proliferator-activated receptors (PPARs) (26, 27). While these receptors have been identified in reproductive tissues, the role of AEA-mediated activity has not yet been clearly elucidated (24, 28, 29).

AEA and 2-AG stimulate PGE_2_ production by fetal gestational membranes (30). In human placentas obtained at term, CP55940 (an agonist) stimulation of CB_1_R resulted in a significant increase in PGE_2_ by the amnion and chorion but a decrease in PGE_2_ in the decidua (30). This increase in PGE_2_ occurred through induction of COX-2 expression in amnion and chorion (30).

## Cannabinoid Receptor Influence in Pregnancy and Labor

CB_1_R and CB_2_R are expressed in human uterine and placental tissue as outlined in **Table 1**. Dennedy and colleagues studied uterine contractility in segments of human myometrial tissue from the upper midline portion of the lower uterine segment obtained from elective cesarean delivery at term (31). They identified the presence of CB_1_R and CB_2_R mRNA in uterine tissue and observed a relaxant effect of AEA and Δ^9^ tetrahydrocannabinol (Δ^9^ THC) on myometrium. Using CB_1_R and CB_2_R antagonists (SR141716 and SR144528), the relaxant effect was determined to be CB_1_R-mediated.

**Table 1:** ECS components in human tissue mid- to late-trimester.

Author	ECS Component	Tissue Type	Experimental Method
**Dennedy, et al.** (31)	CB1R	Uterus	RT-PCR
	CB2R	Uterus	RT-PCR
**Park, et al.** (32)	CB1R	Placenta	IHC
	FAAH	Placenta	IHC
**Acone, et al.** (33)	CB1R	Placenta	Western blot, IHC
**Fugedi, et al.** (34)	CB1R	Placenta	Western blot, IHC
	CB2R	Placenta	Western blot, IHC
	FAAH	Placenta	Western blot, IHC
**Aban, et al.** (35)	CB1R	Placenta	Western blot, IHC
	NAPE-PLD	Placenta	Western blot, IHC
	FAAH	Placenta	Western blot, IHC
**Torella, et al.** (36)	CB1R	Placenta	qPCR
	CB2R	Placenta	qPCR
	NAPE-PLD	Placenta	qPCR
	FAAH	Placenta	qPCR
	MAGL	Placenta	qPCR
	DAGL	Placenta	qPCR
**Kozakiewicz, et al.** (37)	CB1R	Uterus	Western blot, qPCR, IHC
	CB1R	Placenta	Western blot, qPCR
	CRIP1a	Uterus	Western blot, qPCR
	CRIP1a	Placenta	Western blot, qPCR

In the term human placenta, immunodetectable CB_1_R was localized to both cytotrophoblasts and syncytiotrophoblasts (32). Acone and colleagues used immunohistochemistry and western blot to evaluate CB_1_R and FAAH in placentas obtained from laboring and non-laboring subjects (33). They found lower protein density and lesser staining of CB_1_R in placentas obtained from laboring subjects compared to non-laboring subjects (33). They postulated that AEA up-regulates CB_1_R to maintain uterine quiescence and that less availability of CB_1_R is associated with labor. Our group examined CB_1_R and CRIP1a in human uterine and placental tissue obtained during cesarean deliveries, and we found a significant reduction in CB_1_R protein in uterine tissue obtained during labor compared to non-labor. Torrela and colleagues evaluated CB_1_R, CB_2_R, transient receptor potential vanilloid receptor type 1 (TRPV1), FAAH, NAPE-PLD, monoacylglycerol lipase (MAGL) and diacylglycerol lipase (DAGL) in human placental samples obtained after spontaneous vaginal deliveries using qPCR (36). Compared to samples obtained at 30 weeks gestation (preterm), there was a significant increase in CB_1_R mRNA at term. The authors also found a significant increase in the NAPE-PLD/FAAH ratio, thus concluding there to be an increase in placental AEA synthesis at term.

Wang and colleagues used a murine model to evaluate the effect of CB_1_R on parturition (38). They found that CB_1_R knock-out in mice correlated with the early onset of labor and an early rise in corticotrophin-releasing hormone. In wild type mice, CB_1_R silencing in late gestation resulted in labor (38). They correlated these findings with serum levels of progesterone and estradiol. CB_1_R deficient mice were observed to have an early decrease in serum progesterone and increase in serum estradiol levels. Sun and colleagues investigated the effects of sustained AEA signaling in a murine model of lipopolysaccharide (LPS)-induced preterm labor (39). FAAH-knockout mice and wild-type mice that received meth-AEA experienced premature decidual senescence through CB_1_R-mediated activation of p38 signaling (39).

Also using a murine model of LPS-induced preterm labor, Bariani and colleagues examined the effects of LPS on several ECS components (40). A significant increase in uterine NAPE-PLD mRNA and protein in LPS-treated mice was observed, with no significant difference in FAAH activity (40). CB_1_R and CB_2_R were identified in the uterus and there was no change in protein expression between days 13 and 19 of gestation (40). There was, however, more CB_1_R protein and less CB_2_R mRNA identified in LPS-treated mice. LPS treatment did not lead to significant changes in serum progesterone levels. Not surprisingly, the authors found an increase in uterine PGF_2α_ levels in LPS-treated mice. Experiments performed to determine the mechanism for this increase in PGF_2α_ showed it to be a result of AEA acting on CB_1_R (40).

## Prostaglandins and Prostamides

PG production in term pregnancy and initiation of labor is stimulated by pro-inflammatory cytokines which induce COX-2 and myometrial stretch signals (41, 42). AEA and other CB_1_R agonists also induced COX-2 expression and PGE_2_ production in cultured fetal amnion and chorion explants (30, 43). FAAH, expressed in human term placenta (32), metabolizes AEA to contribute the arachidonic acid substrate for COX enzymes. Additionally, AEA itself can be oxidized by COX-2 (but not COX1) to PGH2-EA and the subsequent ethanolamides of PGs, referred to as prostamides (44, 45). AEA can also be oxidized by 5-, 12-, or 15-lipoxygenases to produce their respective OH-AEAs (46), and by cytochrome P450’s to produce 5,6-epoxyeicosatrienoic acid ethanolamide (5,6-EET-EA) (45, 47).

It is now recognized that the radioimmunoassays used to quantitate PGs also recognized prostamides, and that the two classes can be distinguished using liquid chromatography-mass spectrometry (48, 49). This suggests that we need to re-evaluate research findings that attribute physiological processes associated with parturition to PGs, with a new understanding that some of these effects may be due to prostamides. Mitchell and colleagues found that pro-inflammatory cytokines (TNFα, IL-1β) preferentially stimulated PG over prostamide synthesis in human term non-laboring placental choriodecidual tissues (49), and that in amnion tissue explants, IL-1β was particularly efficacious at promoting PGE_2_ synthesis (50). These results are consistent with findings that amniotic fluid PGs were greater in women in spontaneous labor compared with those delivering without labor (50). In that same study, spontaneous labor amniotic fluid prostamides were lower in women with clinical chorioamnionitis compared with undiseased women. Thus, we can speculate that inflammatory responses to AEA in these tissues is dependent upon FAAH to hydrolyze AEA to arachidonic acid to serve as the substrate for COX-2. In contrast, Fonseca and colleagues noted that AEA promoted apoptosis in cultured rat decidual cells (51). In these cells, AEA stimulated MAPK P38 phosphorylation and disinhibition of the NF-κB to induce COX-2, which subsequently used AEA as a substrate to produce prostamide E_2_ (43). Prostamide E_2_, not PGE_2_, was the COX-2 substrate that initiated the intrinsic apoptosis pathway and reduced cell viability (43).

## Discussion

The complex mechanisms that normally convert the uterus from a quiescent to an active contractile state remain unclear. The significant decrease in circulating progesterone that initiates labor in most laboratory animals does not occur in humans (52). The available evidence supports that the active contractile transition involves cessation of the inhibitory effects of progesterone and the activation of estrogen production leading to up-regulation of genes and proteins that enhance uterine contractility (52). There are two functionally distinct progesterone receptors, termed progesterone receptor A (PR-A) and progesterone receptor B (PR-B). PR-B signaling functions to promote activation of genes and proteins that enhance uterine relaxation whereas PR-A represses them (53). Sex steroid hormones are known to influence the expression of components of the ECS in various tissues (9, 54, 55). Estradiol influences expression of CB_1_R and AEA production and degradation in the brain (56). AEA interferes with aromatase transcription and estradiol production in human endometrial stromal cells and human decidual fibroblasts (57). Progesterone increases FAAH activity and expression in human lymphocytes but does not influence CB_1_R (55). Abnormal fluctuations in serum progesterone and estradiol levels are apparent in CB_1_R deficient mice (38). As reviewed by Karasu and colleagues, the termed “endocannabinoid-hormone-cytokine network” plays a significant role in implantation and early pregnancy events (10). It is therefore reasonable to theorize that, based on the interconnections between PGs/prostamides, sex steroid hormones and endocannabinoids, the ECS is likely to have a meaningful part in pregnancy maintenance and timing of labor.

Studies evaluating the effects of cannabis use in pregnancy have provided mixed results (58, 59). They are limited by confounding factors (polysubstance abuse, tobacco use), the reliance on subject self-reporting, and the perplexity of obtaining a reliable biologic sample for drug testing. Cannabis use in pregnancy has been associated with increased risk of spontaneous preterm birth (60), stillbirth (61), poor fetal growth (59), and adverse neonatal outcomes (62). Data are limited regarding potential effects of marijuana on labor itself. In one of the few studies evaluating labor patterns in marijuana users, Greenland and colleagues found that subjects reporting marijuana use had a higher risk of experiencing prolonged, arrested or precipitous labor (63). Many of the available data regarding cannabis use in pregnancy were collected prior to the decriminalization of marijuana, the introduction of newer methods of cannabis consumption and the introduction of higher potency compounds. Given the increasing prevalence of cannabis use in pregnancy (58, 64), it is imperative to not only evaluate the risks of marijuana use in pregnancy but also to gain a better understanding of the mechanisms by which the ECS contributes to pregnancy maintenance and labor.

AEA is degraded primarily by FAAH to produce arachidonic acid and ethanolamine (16). However, AEA oxidization by COX-2 to PG-ethanolamides (prostamides) (16) highlights a significant overlap between the ECS and PG production. PGs are routinely used in obstetrics for induction of labor and the treatment of postpartum hemorrhage related to uterine atony. Indomethacin, a non-selective COX inhibitor, is one of the recommended first-line tocolytic therapies for preterm labor (18). Indomethacin has been recently identified to be a positive allosteric modulator of CB_1_R (65). Its modulating effects enhance AEA-dependent binding, β-arrestin 1 recruitment, cAMP inhibition and ERK1/2 phosphorylation (65). Bariani and colleagues found that LPS-induced preterm labor in a murine model correlates with increased CB_1_R expression and, even without the addition of LPS, administration of AEA resulted in a CB_1_R-mediated increase in PGF_2α_ (40). In contrast, in a murine model without LPS administration, earlier onset of labor was identified in mice lacking CB_1_R (38). Although there are limitations to this based on the differences between rodent and human labor, this highlights the possibility that ECS expression may differ in infection-related labor compared to normal term labor.

Although an initial study utilizing AEA and/or PEA as a biomarker in the risk assessment for preterm birth is promising, that evaluation was limited to a population with a higher-risk of preterm birth (21). Additional studies including a more generalized and larger population are needed. The current standard of care involving measurement of cervical length and, in some cases, cervicovaginal fetal fibronectin does not reliably predict preterm birth (18). Additionally, assessment of cervical length requires equipment (ultrasound with transvaginal probe) and personnel with adequate training and who are readily available to perform the exam. A blood test would be more feasible in many situations.

Significant racial disparities exist in the rate of preterm birth (66). This racial disparity persists when evaluating women with similar socioeconomic status (67), leading many to believe that genetic variation may play a role. A cross-sectional study of 667 subjects identified racial differences in CNR1 and FAAH polymorphisms associated with obesity (68). Given the increasing evidence of the ECS involvement in normal and abnormal pregnancy outcomes, genetic variation among the components of the ECS pertaining to abnormal pregnancy outcomes should be explored.

The biology of labor is complex and includes interplay among steroid hormones, cytokines and PGs affecting the maternal-fetal interface (12, 13, 69). There exists a significant overlap between the inflammatory pathway, steroid hormones and endocannabinoids (10). Because the ECS modulates metabolic and inflammatory cell signaling and can modulate cell differentiation, cell proliferation and cell death, it is reasonable to expect that the ECS exerts an influence on the regulation of labor. More research is needed for significant conclusions regarding ECS specific role in pregnancy maintenance and the timing of labor.

## Author Contributions

The authors confirm contribution to the manuscript as follows: MK, CG and AH critically reviewed the literature. MK drafted the article. MK, CG and AH reviewed and revised the manuscript. All authors contributed to the article and approved the submitted version.

## Funding

This work was supported by National Institute on Drug Abuse (NIDA) grant R01-DA042157 and Eunice Kennedy Shriver National Institute of Child Health and Human Development (NICHD) grant R01-HD096385.

## Conflict of Interest

The author CG is the Chief Medical Officer of Nixxi (https://nixxihealth.com), a company developing preterm birth prediction tools.

The remaining authors declare that the research was conducted in the absence of any commercial or financial relationships that could be construed as a potential conflict of interest.

## Abbreviations

2-AG, 2-arachidonoylglycerol; AEA, anandamide; CB1R, CB1 cannabinoid receptor; CB2R, CB2 cannabinoid receptor; COX-2, cyclooxygenase-2; CRIP1a, cannabinoid receptor interacting protein 1a; ECS, endocannabinoid cell-signaling system; FAAH, fatty acid amide hydrolase; MAPK, mitogen-activated protein kinase; OTR, oxytocin receptor; PG, prostaglandin; PEA, palmitylethanolamide; OEA, oleoylethanolamide; NAPE-PLD, N-acylphosphatidylethanolamine-specific phospholipase D; meth-AEA, methanandamide; cAMP, cyclic adenosine monophosphate; PKA, protein kinase A; Δ9 THC, Δ9 tetrahydrocannabinol; TRPV1, transient receptor potential vanilloid receptor type 1; MAGL, monoacylglycerol lipase; DAGL, diacylglycerol lipase; LPS, lipopolysaccharide; 5,6-EET-EA, 5,6-epoxyeicosatrienoic acid ethanolamide; PR-A, progesterone receptor A; PR-B, progesterone receptor B; IHC, immunohistochemistry.

## References

[1] Maccarrone M Bab I Bíró T Cabral GA Dey SK Di Marzo V. Endocannabinoid Signaling at the Periphery: 50 Years After THC. Trends Pharmacol Sci (2015) 36(5):277–96. 10.1016/j.tips.2015.02.008 PMC4420685 25796370

[2] Howlett AC Blume LC Dalton GD. CB(1) Cannabinoid Receptors and Their Associated Proteins. Curr Med Chem (2010) 17(14):1382–93. 10.2174/092986710790980023 PMC3179980 20166926

[3] Howlett AC. The Cannabinoid Receptors. Prostaglandins Other Lipid Mediat (2002) 68-69:619–31. 10.1016/S0090-6980(02)00060-6 12432948

[4] Booth WT Walker NB Lowther WT Howlett AC. Cannabinoid Receptor Interacting Protein 1a (CRIP1a): Function and Structure. Molecules (2019) 24(20):3672. 10.3390/molecules24203672 PMC6832298 31614728

[5] Trabucco E Acone G Marenna A Pierantoni R Cacciola G Chioccarelli T. Endocannabinoid System in First Trimester Placenta: Low FAAH and High CB1 Expression Characterize Spontaneous Miscarriage. Placenta (2009) 30(6):516–22. 10.1016/j.placenta.2009.03.015 19419760

[6] Meccariello R Battista N Bradshaw HB Wang H. Updates in Reproduction Coming From the Endocannabinoid System. Int J Endocrinol (2014) 2014:412354. 10.1155/2014/412354 24550985 PMC3914453

[7] Sun X Dey SK. Endocannabinoid Signaling in Female Reproduction. ACS Chem Neurosci (2012) 3(5):349–55. 10.1021/cn300014e PMC3382454 22860202

[8] Correa F Wolfson ML Valchi P Aisemberg J Franchi AM. Endocannabinoid System and Pregnancy. Reproduction (2016) 152(6):R191–200. 10.1530/REP-16-0167 27798285

[9] Santoro A Mele E Marino M Viggiano A Nori SL Meccariello R. The Complex Interplay Between Endocannabinoid System and the Estrogen System in Central Nervous System and Periphery. Int J Mol Sci (2021) 22(2):972. 10.3390/ijms22020972 33478092 PMC7835826

[10] Karasu T Marczylo TH Maccarrone M Konje JC. The Role of Sex Steroid Hormones, Cytokines and the Endocannabinoid System in Female Fertility. Hum Reprod Update (2011) 17(3):347–61. 10.1093/humupd/dmq058 21227997

[11] Maia J Fonseca BM Teixeira N Correia-da-Silva G. The Fundamental Role of the Endocannabinoid System in Endometrium and Placenta: Implications in Pathophysiological Aspects of Uterine and Pregnancy Disorders. Hum Reprod Update (2020) 26(4):586–602. 10.1093/humupd/dmaa005 32347309 PMC7317288

[12] Renthal NE Williams KC Montalbano AP Chen CC Gao L Mendelson CR. Molecular Regulation of Parturition: A Myometrial Perspective. Cold Spring Harb Perspect Med (2015) 5(11):a023069. 10.1101/cshperspect.a023069 26337112 PMC4632865

[13] Romero R Dey SK Fisher SJ. Preterm Labor: One Syndrome, Many Causes. Science (2014) 345(6198):760–5. 10.1126/science.1251816 PMC4191866 25124429

[14] Habayeb OM Taylor AH Evans MD Cooke MS Taylor DJ Bell SC. Plasma Levels of the Endocannabinoid Anandamide in Women–a Potential Role in Pregnancy Maintenance and Labor? J Clin Endocrinol Metab (2004) 89(11):5482–7. 10.1210/jc.2004-0681 15531501

[15] Nallendran V Lam PM Marczylo TH Bankart MJ Taylor AH Taylor DJ. The Plasma Levels of the Endocannabinoid, Anandamide, Increase With the Induction of Labour. BJOG (2010) 117(7):863–9. 10.1111/j.1471-0528.2010.02555.x 20406230

[16] Maccarrone M. Metabolism of the Endocannabinoid Anandamide: Open Questions After 25 Years. Front Mol Neurosci (2017) 10:166. 10.3389/fnmol.2017.00166 28611591 PMC5447297

[17] Maccarrone M Bari M Battista N Finazzi-Agrò A. Estrogen Stimulates Arachidonoylethanolamide Release From Human Endothelial Cells and Platelet Activation. Blood (2002) 100(12):4040–8. 10.1182/blood-2002-05-1444 12393387

[18] Gynecologists ACoOa Bulletins—Obstetrics CoP. ACOG Practice Bulletin No. 127: Management of Preterm Labor. Obstet Gynecol (2012) 119(6):1308–17. 10.1097/AOG.0b013e31825af2f0 22617615

[19] Rose CH McWeeney DT Brost BC Davies NP Watson WJ. Cost-Effective Standardization of Preterm Labor Evaluation. Am J Obstet Gynecol (2010) 203(3):250.e1–5. 10.1016/j.ajog.2010.06.037 20816147

[20] McPheeters ML Miller WC Hartmann KE Savitz DA Kaufman JS Garrett JM. The Epidemiology of Threatened Preterm Labor: A Prospective Cohort Study. Am J Obstet Gynecol (2005) 192(4):1325–9; discussion 9-30. 10.1016/j.ajog.2004.12.055 15846230

[21] Bachkangi P Taylor AH Bari M Maccarrone M Konje JC. Prediction of Preterm Labour From a Single Blood Test: The Role of the Endocannabinoid System in Predicting Preterm Birth in High-Risk Women. Eur J Obstet Gynecol Reprod Biol (2019) 243:1–6. 10.1016/j.ejogrb.2019.09.029 31618675

[22] Accialini P Etcheverry T Malbrán MN Leguizamón G Maté S Farina M. Anandamide Regulates Oxytocin/Oxytocin Receptor System in Human Placenta at Term. Placenta (2020) 93:23–5. 10.1016/j.placenta.2020.02.012 32090965

[23] Yulia A Singh N Lei K Sooranna SR Johnson MR. Cyclic AMP Effectors Regulate Myometrial Oxytocin Receptor Expression. Endocrinology (2016) 157(11):4411–22. 10.1210/en.2016-1514 27673556

[24] Brighton PJ McDonald J Taylor AH Challiss RA Lambert DG Konje JC. Characterization of Anandamide-Stimulated Cannabinoid Receptor Signaling in Human ULTR Myometrial Smooth Muscle Cells. Mol Endocrinol (2009) 23(9):1415–27. 10.1210/me.2009-0097 PMC2737560 19477951

[25] Smart D Gunthorpe MJ Jerman JC Nasir S Gray J Muir AI. The Endogenous Lipid Anandamide Is a Full Agonist at the Human Vanilloid Receptor (Hvr1). Br J Pharmacol (2000) 129(2):227–30. 10.1038/sj.bjp.0703050 PMC1571834 10694225

[26] Russo R LoVerme J La Rana G D’Agostino G Sasso O Calignano A. Synergistic Antinociception by the Cannabinoid Receptor Agonist Anandamide and the PPAR-Alpha Receptor Agonist GW7647. Eur J Pharmacol (2007) 566(1-3):117–9. 10.1016/j.ejphar.2007.03.007 PMC1997313 17434479

[27] Bouaboula M Hilairet S Marchand J Fajas L Le Fur G Casellas P. Anandamide Induced PPARgamma Transcriptional Activation and 3T3-L1 Preadipocyte Differentiation. Eur J Pharmacol (2005) 517(3):174–81. 10.1016/j.ejphar.2005.05.032 15987634

[28] Dong K Zhang MX Liu Y Su XL Chen B Zhang XL. Peroxisome Proliferator-Activated Receptor Alpha Expression Changes in Human Pregnant Myometrium. Reprod Sci (2013) 20(6):654–60. 10.1177/1933719112461187 PMC3713545 23144166

[29] Bogacka I Kurzynska A Bogacki M Chojnowska K. Peroxisome Proliferator-Activated Receptors in the Regulation of Female Reproductive Functions. Folia Histochem Cytobiol (2015) 53(3):189–200. 10.5603/fhc.a2015.0023 26339984

[30] Mitchell MD Sato TA Wang A Keelan JA Ponnampalam AP Glass M. Cannabinoids Stimulate Prostaglandin Production by Human Gestational Tissues Through a Tissue- and CB1-Receptor-Specific Mechanism. Am J Physiol Endocrinol Metab (2008) 294(2):E352–6. 10.1152/ajpendo.00495.2007 18042663

[31] Dennedy MC Friel AM Houlihan DD Broderick VM Smith T Morrison JJ. Cannabinoids and the Human Uterus During Pregnancy. Am J Obstet Gynecol (2004) 190(1):2–9; discussion 3A. 10.1016/j.ajog.2003.07.013 14749627

[32] Park B Gibbons HM Mitchell MD Glass M. Identification of the CB1 Cannabinoid Receptor and Fatty Acid Amide Hydrolase (FAAH) in the Human Placenta. Placenta (2003) 24(10):990–5. 10.1016/S0143-4004(03)00165-6 12744923

[33] Acone G Trabucco E Colacurci N Cobellis L Mackie K Meccariello R. Low Type I Cannabinoid Receptor Levels Characterize Placental Villous in Labouring Delivery. Placenta (2009) 30(2):203–5. 10.1016/j.placenta.2008.11.018 19097644

[34] Fügedi G Molnár M Rigó J Schönléber J Kovalszky I Molvarec A. Increased Placental Expression of Cannabinoid Receptor 1 in Preeclampsia: An Observational Study. BMC Pregnancy Childbirth (2014) 14:395. 10.1186/s12884-014-0395-x 25444073 PMC4264532

[35] Abán C Leguizamón GF Cella M Damiano A Franchi AM Farina MG. Differential Expression of Endocannabinoid System in Normal and Preeclamptic Placentas: Effects on Nitric Oxide Synthesis. Placenta (2013) 34(1):67–74. 10.1016/j.placenta.2012.10.009 23122699

[36] Torella M Bellini G Punzo F Argenziano M Schiattarella A Labriola D. Tnf-α Effect on Human Delivery Onset by CB1/TRPV1 Crosstalk: New Insights Into Endocannabinoid Molecular Signaling in Preterm vs. Term Labor. Analysis of the EC/EV Pathway and Predictive Biomarkers for Early Diagnosis of Preterm Delivery. Minerva Ginecol (2019) 71(5):359–64. 10.23736/S0026-4784.19.04405-8 31698890

[37] Kozakiewicz ML Zhang J Leone-Kabler S Yamaleyeva LM McDonald AG Brost BC. Differential Expression of CB1 Cannabinoid Receptor and Cannabinoid Receptor Interacting Protein 1a in Labor. Cannabis Cannabinoid Res (2021). 10.1089/can.2020.0107 PMC9225407 33998898

[38] Wang H Xie H Dey SK. Loss of Cannabinoid Receptor CB1 Induces Preterm Birth. PloS One (2008) 3(10):e3320. 10.1371/journal.pone.0003320 18833324 PMC2553193

[39] Sun X Deng W Li Y Tang S Leishman E Bradshaw HB. Sustained Endocannabinoid Signaling Compromises Decidual Function and Promotes Inflammation-Induced Preterm Birth. J Biol Chem (2016) 291(15):8231–40. 10.1074/jbc.M115.707836 PMC4825023 26900150

[40] Bariani MV Domínguez Rubio AP Cella M Burdet J Franchi AM Aisemberg J. Role of the Endocannabinoid System in the Mechanisms Involved in the LPS-Induced Preterm Labor. Reproduction (2015) 150(6):463–72. 10.1530/REP-15-0211 26347521

[41] Gibb W. The Role of Prostaglandins in Human Parturition. Ann Med (1998) 30(3):235–41. 10.3109/07853899809005850 9677008

[42] Keelan JA Blumenstein M Helliwell RJ Sato TA Marvin KW Mitchell MD. Cytokines, Prostaglandins and Parturition‐A Review. Placenta (2003) 24(Suppl A):S33–46. 10.1053/plac.2002.0948 12842412

[43] Almada M Piscitelli F Fonseca BM Di Marzo V Correia-da-Silva G Teixeira N. Anandamide and Decidual Remodelling: COX-2 Oxidative Metabolism as a Key Regulator. Biochim Biophys Acta (2015) 1851(11):1473–81. 10.1016/j.bbalip.2015.08.011 26335727

[44] Kozak KR Prusakiewicz JJ Marnett LJ. Oxidative Metabolism of Endocannabinoids by COX-2. Curr Pharm Des (2004) 10(6):659–67. 10.2174/1381612043453081 14965328

[45] Rouzer CA Marnett LJ. Non-Redundant Functions of Cyclooxygenases: Oxygenation of Endocannabinoids. J Biol Chem (2008) 283(13):8065–9. 10.1074/jbc.R800005200 PMC2417164 18250160

[46] van der Stelt M van Kuik JA Bari M van Zadelhoff G Leeflang BR Veldink GA. Oxygenated Metabolites of Anandamide and 2-Arachidonoylglycerol: Conformational Analysis and Interaction With Cannabinoid Receptors, Membrane Transporter, and Fatty Acid Amide Hydrolase. J Med Chem (2002) 45(17):3709–20. 10.1021/jm020818q 12166944

[47] Zelasko S Arnold WR Das A. Endocannabinoid Metabolism by Cytochrome P450 Monooxygenases. Prostaglandins Other Lipid Mediat (2015) 116-117:112–23. 10.1016/j.prostaglandins.2014.11.002 25461979

[48] Glass M Hong J Sato TA Mitchell MD. Misidentification of Prostamides as Prostaglandins. J Lipid Res (2005) 46(7):1364–8. 10.1194/jlr.C500006-JLR200 15863842

[49] Mitchell MD Rice GE Vaswani K Kvaskoff D Peiris HN. Differential Regulation of Eicosanoid and Endocannabinoid Production by Inflammatory Mediators in Human Choriodecidua. PloS One (2016) 11(2):e0148306. 10.1371/journal.pone.0148306 26840435 PMC4740432

[50] Peiris HN Vaswani K Holland O Koh YQ Almughlliq FB Reed S. Altered Productions of Prostaglandins and Prostamides by Human Amnion in Response to Infectious and Inflammatory Stimuli Identified by Mutliplex Mass Spectrometry. Prostaglandins Leukot Essent Fatty Acids (2020) 154:102059. 10.1016/j.plefa.2020.102059 32014738

[51] Fonseca BM Correia-da-Silva G Teixeira NA. Anandamide-Induced Cell Death: Dual Effects in Primary Rat Decidual Cell Cultures. Placenta (2009) 30(8):686–92. 10.1016/j.placenta.2009.05.012 19560819

[52] Norwitz ER Mahendroo M Lye SJ. Creasy and Resnik’s Maternal-Fetal Medicine: Principles and Practice. 8th Edition. Resnik R Lockwood CJ Moore TR Greene MF Copel JA Silver RM, editors. Philadelphia, PA: Elsevier (2019).

[53] Peters GA Yi L Skomorovska-Prokvolit Y Patel B Amini P Tan H. Inflammatory Stimuli Increase Progesterone Receptor-A Stability and Transrepressive Activity in Myometrial Cells. Endocrinology (2017) 158(1):158–69. 10.1210/en.2016-1537 PMC5412979 27886516

[54] MacCarrone M De Felici M Bari M Klinger F Siracusa G Finazzi-Agrò A. Down-Regulation of Anandamide Hydrolase in Mouse Uterus by Sex Hormones. Eur J Biochem (2000) 267(10):2991–7. 10.1046/j.1432-1033.2000.01316.x 10806398

[55] Maccarrone M Valensise H Bari M Lazzarin N Romanini C Finazzi-Agrò A. Progesterone Up-Regulates Anandamide Hydrolase in Human Lymphocytes: Role of Cytokines and Implications for Fertility. J Immunol (2001) 166(12):7183–9. 10.4049/jimmunol.166.12.7183 11390466

[56] González S Bisogno T Wenger T Manzanares J Milone A Berrendero F. Sex Steroid Influence on Cannabinoid CB(1) Receptor mRNA and Endocannabinoid Levels in the Anterior Pituitary Gland. Biochem Biophys Res Commun (2000) 270(1):260–6. 10.1006/bbrc.2000.2406 10733937

[57] Almada M Oliveira A Amaral C Fernandes PA Ramos MJ Fonseca B. Anandamide Targets Aromatase: A Breakthrough on Human Decidualization. Biochim Biophys Acta Mol Cell Biol Lipids (2019) 1864(12):158512. 10.1016/j.bbalip.2019.08.008 31454668

[58] Metz TD Borgelt LM. Marijuana Use in Pregnancy and While Breastfeeding. Obstet Gynecol (2018) 132(5):1198–210. 10.1097/AOG.0000000000002878 PMC6370295 30234728

[59] Conner SN Bedell V Lipsey K Macones GA Cahill AG Tuuli MG. Maternal Marijuana Use and Adverse Neonatal Outcomes: A Systematic Review and Meta-Analysis. Obstet Gynecol (2016) 128(4):713–23. 10.1097/AOG.0000000000001649 27607879

[60] Leemaqz SY Dekker GA McCowan LM Kenny LC Myers JE Simpson NA. Maternal Marijuana Use Has Independent Effects on Risk for Spontaneous Preterm Birth But Not Other Common Late Pregnancy Complications. Reprod Toxicol (2016) 62:77–86. 10.1016/j.reprotox.2016.04.021 27142189

[61] Varner MW Silver RM Rowland Hogue CJ Willinger M Parker CB Thorsten VR. Association Between Stillbirth and Illicit Drug Use and Smoking During Pregnancy. Obstet Gynecol (2014) 123(1):113–25. 10.1097/AOG.0000000000000052 PMC3931517 24463671

[62] Metz TD Allshouse AA Hogue CJ Goldenberg RL Dudley DJ Varner MW. Maternal Marijuana Use, Adverse Pregnancy Outcomes, and Neonatal Morbidity. Am J Obstet Gynecol (2017) 217(4):478.e1–.e8. 10.1016/j.ajog.2017.05.050 PMC5614818 28578174

[63] Greenland S Staisch KJ Brown N Gross SJ. The Effects of Marijuana Use During Pregnancy. I. A Preliminary Epidemiologic Study. Am J Obstet Gynecol (1982) 143(4):408–13. 10.1016/0002-9378(82)90082-5 7091206

[64] Brown QL Sarvet AL Shmulewitz D Martins SS Wall MM Hasin DS. Trends in Marijuana Use Among Pregnant and Nonpregnant Reproductive-Aged Women, 2002-2014. JAMA (2017) 317(2):207–9. 10.1001/jama.2016.17383 PMC5595220 27992619

[65] Laprairie RB Mohamed KA Zagzoog A Kelly MEM Stevenson LA Pertwee R. Indomethacin Enhances Type 1 Cannabinoid Receptor Signaling. Front Mol Neurosci (2019) 12:257. 10.3389/fnmol.2019.00257 31680861 PMC6813218

[66] Manuck TA. Racial and Ethnic Differences in Preterm Birth: A Complex, Multifactorial Problem. Semin Perinatol (2017) 41(8):511–8. 10.1053/j.semperi.2017.08.010 PMC6381592 28941962

[67] Collins JW David RJ Simon DM Prachand NG. Preterm Birth Among African American and White Women With a Lifelong Residence in High-Income Chicago Neighborhoods: An Exploratory Study. Ethn Dis (2007) 17(1):113–7. 17274219

[68] Thethi TK Sigel A Japa S Katalenich B Liu S Nguyen T. Racial and Sex Differences in the Polymorphisms of the Endocannabinoid Receptor Genes in Obesity. J Diabetes Complications (2020) 34(11):107682. 10.1016/j.jdiacomp.2020.107682 32732136 PMC7508856

[69] Olson DM. The Role of Prostaglandins in the Initiation of Parturition. Best Pract Res Clin Obstet Gynaecol (2003) 17(5):717–30. 10.1016/S1521-6934(03)00069-5 12972010

